# Advanced susceptibility analysis of ground deformation disasters using large language models and machine learning: A Hangzhou City case study

**DOI:** 10.1371/journal.pone.0310724

**Published:** 2024-12-12

**Authors:** Bofan Yu, Huaixue Xing, Weiya Ge, Liling Zhou, Jiaxing Yan, Yun-an Li

**Affiliations:** 1 Nanjing Center, China Geological Survey, Nanjing Center, China Geological Survey, Nanjing, The People’s Republic of China; 2 The Ministry of Natural Resources’ Urban Underground Space Exploration and Evaluation Technology Innovation Center, Qingdao, The People’s Republic of China; 3 Zhejiang Institute of Geosciences, Observation and Research Station of Zhejiang Coastal Urban Geological Security, Ministry of Natural Resources, Hangzhou, The People’s Republic of China; 4 The Institute of Geological Survey of China University of Geosciences (Wuhan), China University of Geosciences (Wuhan), The People’s Republic of China; Istanbul University: Istanbul Universitesi, TÜRKIYE

## Abstract

To address the prevailing scenario where comprehensive susceptibility assessments of ground deformation disasters primarily rely on knowledge-driven models, with weight judgments largely founded on expert subjective assessments, this study initially explores the feasibility of integrating data-driven models into the evaluation of urban ground collapse and subsidence. Hangzhou city, characterized by filled soil and silty sand, was selected as the representative study area. Nine pertinent evaluation factors were identified, and the RF-BP neural network coupling model was employed to assess the susceptibility of ground collapse and subsidence in the study area, the results indicate that the stacked model achieved a 7% increase in AUC value compared to the single model. Subsequently, this study utilized the advanced large language model (LLM), ChatGPT-4, to supplant expert judgment in the weight determination of ground deformation disasters. The advantages of ChatGPT-4, such as its ability to process vast amounts of data and provide consistent, unbiased judgments, were highlighted. ChatGPT-4’s assessments were validated by geological experts in the study area through the analytic hierarchy process. The results show that, by analyzing the same textual materials, the weights determined by experts differed by only 3% from those judged by ChatGPT, demonstrating the reliability and human-expert-like logic of ChatGPT-4’s judgments. Finally, a comprehensive susceptibility assessment of ground deformation disasters was conducted utilizing ChatGPT-4’s judgment results, yielding favorable outcomes.

## Introduction

In geological disaster research, phenomena such as ground subsidence, collapse, and fissures are collectively referred to as ground deformation geological disasters. These phenomena, caused by the compression and displacement of soil and rock due to natural conditions and human factors, lead to ground sinking, collapsing, and cracking, posing hazards to engineering facilities and environments, and endangering lives and properties [1–3]. With the rise of urban underground construction and escalating human activities disturbing strata and structures, ground deformation disasters are becoming more frequent, representing significant threats to people’s lives and property. Therefore, the assessment and prediction of these disasters, especially in densely populated urban areas, is of paramount importance. In Hangzhou, ground deformation disasters predominantly occur in the plains, influenced by a combination of geological, hydrological, and anthropogenic factors. Geologically, these areas feature extensive distributions of sand layers, artificial fill, and Quaternary soft soil layers. Hydrologically, historical extensive groundwater extraction coupled with the abundant groundwater facilitated by ancient river channel distributions significantly impacts the region. Anthropogenically, this region represents Hangzhou’s most economically developed area, characterized by large-scale urban construction, significant engineering loads, and a dense network of underground pipelines. Consequently, the primary ground deformation disasters in Hangzhou manifest as subsidence and collapses, each driven by distinct factors. Subsidence primarily results from extensive urban development and the prevalent distribution of Quaternary soft soil layers, which are susceptible to compression under structural loads. In contrast, collapses are primarily triggered by the presence of artificial fill and the failure of underground pipelines, conditions that create voids and structural weaknesses in the subsurface, leading to sudden ground failures. The evaluation and prediction of geological disasters require a thorough understanding of susceptibility, which not only informs about the geological conditions but also facilitates the proper zoning of the selected area [4–7] Nevertheless, relying solely on assessments of single disaster types may not fully encapsulate the geological safety of a region. Hence, a more comprehensive approach that includes multiple disaster types is often adopted [8–10]. Traditionally, assessments, particularly those addressing subsidence and collapse, have depended on knowledge-driven models like the Analytic Hierarchy Process (AHP) [11–13]. Particularly for collapse-related disasters, which occur over small areas, machine learning-based susceptibility assessments of such disasters are, to our knowledge, currently an unexplored area. This method involves subjective scoring by experts to assign weights to various indicators for susceptibility analysis [14–16]. Also, this approach especially prevalent in the evaluation of collapse-related disasters, which typically affect smaller regions and have not yet been extensively explored through machine learning-based assessments. When assessing the relative weights of different types of disasters within a specific area, the lack of learning samples means that reliance on expert judgment is the only viable option.

The AHP method, although widely used for comprehensive disaster evaluations, increasingly shows its limitations due to its heavy reliance on expert judgments [17]. This dependency introduces subjectivity, potentially affected by emotional states or evaluator fatigue, especially when numerous criteria are involved. Consequently, the scientific community is gradually shifting towards data-driven models, which are adept at processing complex datasets and thereby enhance the accuracy of predictions. Unlike traditional models, data-driven models are capable of handling more complex datasets, thereby improving prediction accuracy. These models have proven particularly effective in analyzing susceptibility to large-scale disasters like landslides and earthquakes [18–22]. Recent innovations have included the integration of various machine learning models, such as combining Back Propagation (BP) neural networks with Support Vector Machines (SVM), to capitalize on the strengths of each model while minimizing the weaknesses, thereby improving overall predictive performance [23,24]. However, despite the advances in data-driven methodologies, the challenge of assigning relative weights to different types of disasters within specific areas remains largely unresolved without extensive historical and geological data. Currently, this aspect of disaster evaluation still often relies on the subjective judgments of experts, underscoring a significant gap that persists in comprehensive disaster risk assessments. This gap highlights the ongoing need for developing methodologies that can integrate large-scale data analysis while accommodating the unique characteristics of various disaster types. Amid the progression of artificial intelligence technology, large language models (LLMs) such as ChatGPT-4 are being increasingly utilized in various fields for data analysis [25–28]. Large language models (LLMs) are AI models crafted to comprehend and produce human language. Trained with extensive text data, they excel in various tasks such as text summarization, translation, sentiment analysis, among others [29]. A distinctive feature of LLMs is their immense scale, encompassing billions of parameters to learn intricate language patterns. These models are generally based on deep learning architectures, such as transformers, facilitating impressive performance in diverse NLP tasks [30,31]. LLMs such as ChatGPT-4 are capable of processing large text datasets, handling complex information quickly and accurately, unencumbered by human biases or fatigue. They can learn from historical data to identify patterns and connections, enabling deeper and more comprehensive analysis. Despite their application in fields such as medicine and biology for data processing and replicating experiments [32–35]. LLMs have yet to be explored in geology for assisting in data processing and professional judgments.

**Fig 1:**
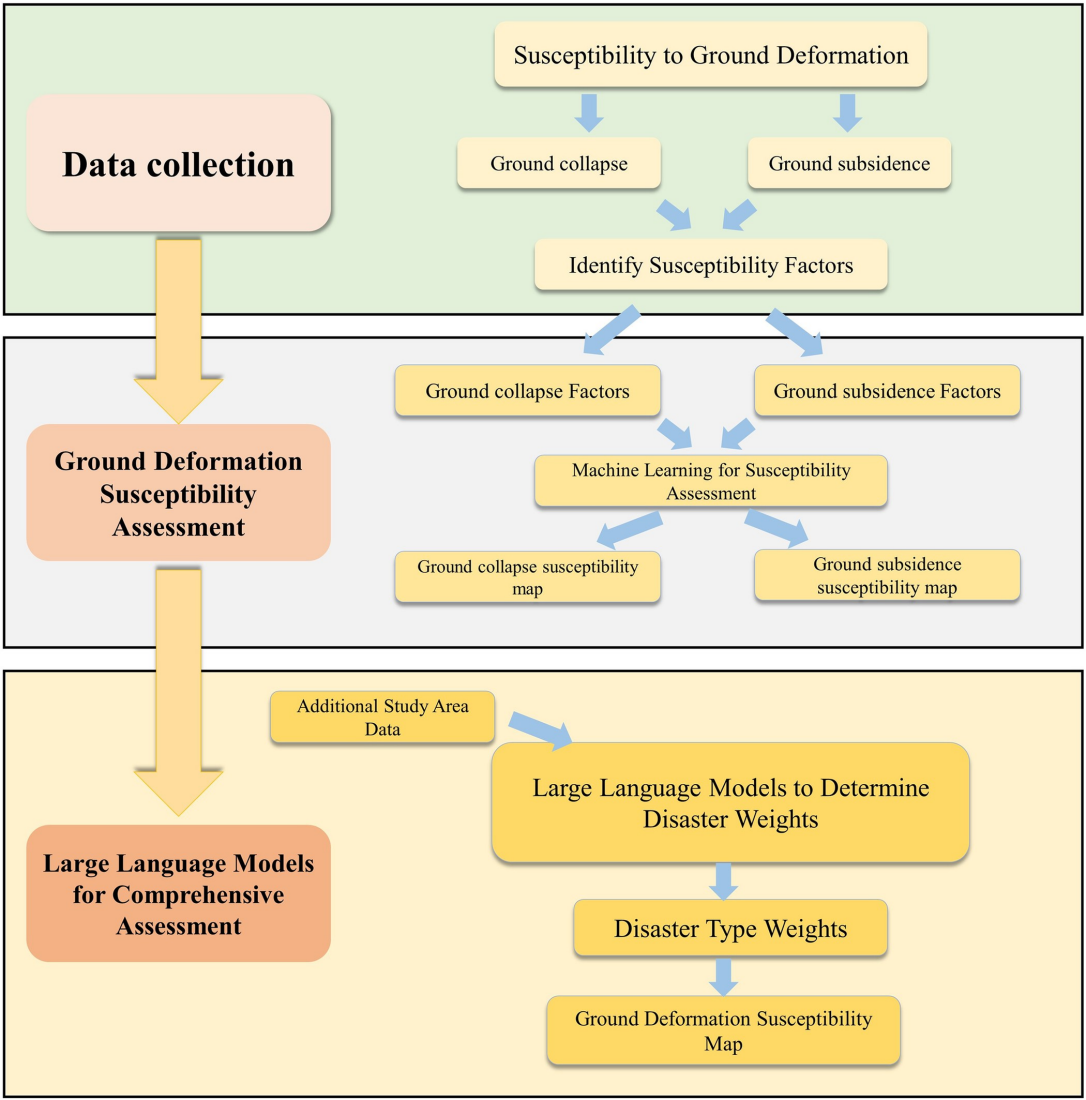
Research flowchart.

This study assesses the susceptibility of ground deformation disasters in commonly filled and silty areas in Hangzhou by employing machine learning models. It investigates the feasibility of utilizing coupled models over single models to enhance evaluation effectiveness, particularly providing a viable template for applying machine learning to small-scale, collapse-type disasters. Additionally, the study introduces an innovative approach by using LLMs for data processing and weight determination in the comprehensive evaluation process. Through comparison with expert AHP evaluations, this research confirms the advantages of LLMs like ChatGPT-4 in fast data processing, consistent judgment, and freedom from human subjective factors, while concurrently analyzing its limitations and contrasting the judgment logic of ChatGPT-4 with that of human experts. This marks the first application of LLM in the field of geological data analysis and judgment, and it also underscores the potential of LLMs in processing data and augmenting or even replacing human judgment in geological fields. Our research process is illustrated in Fig 1.

## Background and methods

### Study area

**Fig 2:**
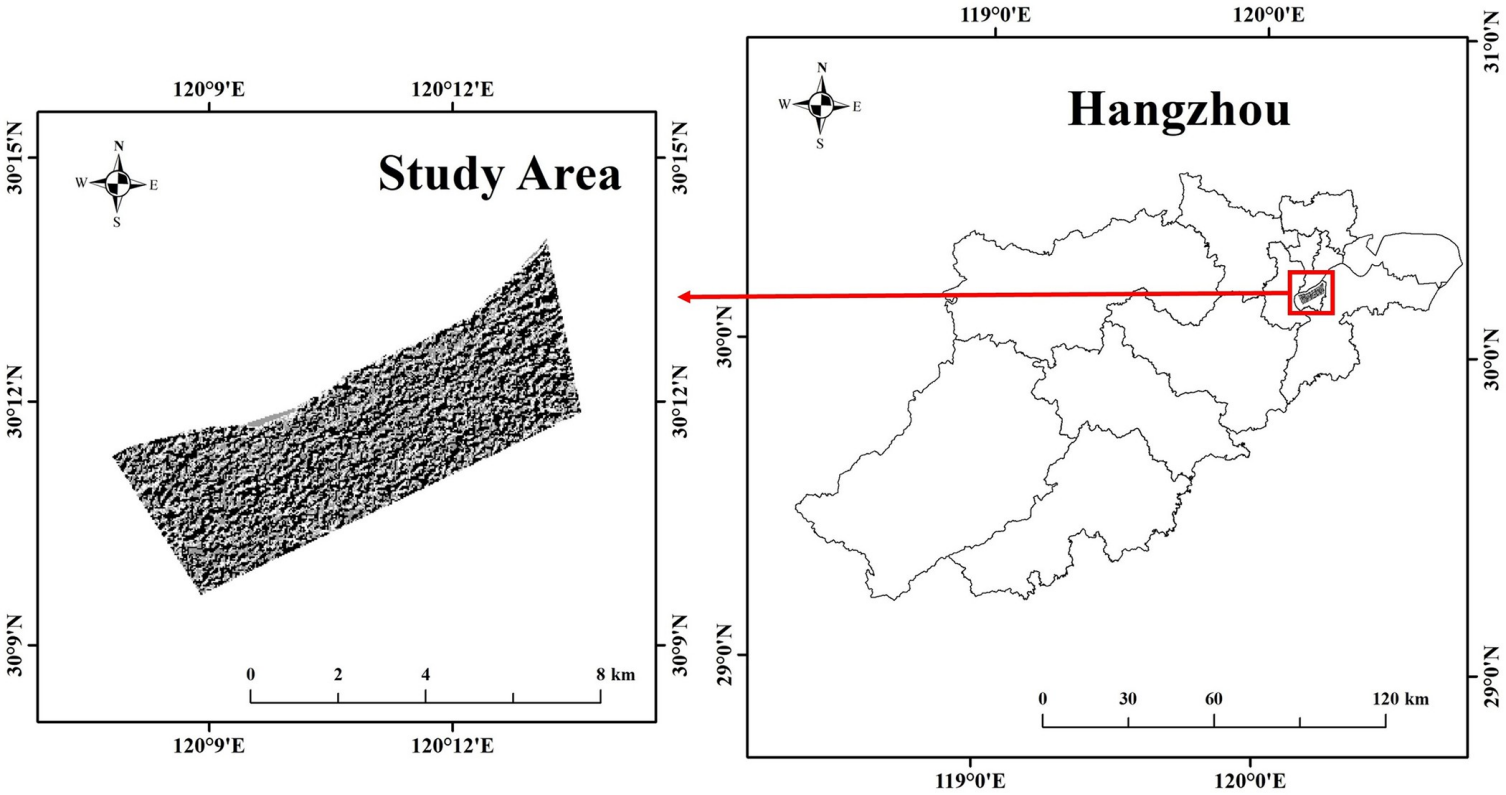
Location of the study area.

The silty clay soil in Hangzhou City predominantly occupies the plain regions. These areas, characterized by accumulative, alluvial, and marine plains, are among the most representative. This study selects the typical filled soil-silty clay region along the southern bank of the Qiantang River in Binjiang District as its focus. We chose this study area with the aim of using it as a model, so that our methods can be extended to the entire Hangzhou region in the future. The study area spans 31.97 km^2^ and includes significant urban infrastructure such as subways, expressways, and elevated roads, indicating substantial human modifications. Geographically, it lies along the western boundary of the Xiaoshao Plain, bordered to the north by the Hangzhou duplex hill and to the south by the Puyang River Plain. The terrain is primarily flat plains interspersed with a few low hills, exhibiting clear geomorphological boundaries, flat topography, and a dense river network. The soil composition mainly includes sandy silty soil and silt, with the sand and gravel layer buried at depths of approximately 35–50 meters. Human activities have significantly influenced the area, with high-rise structures typically utilizing sand-gravel layers or bedrock as their foundational stratum. The sedimentary composition primarily consists of gray to dark gray silty mud clay and silty fine clay, characterized by pronounced horizontal stratification and high-water content. The Quaternary strata in this region are largely from the lower Holocene, formed during the early Fuyang marine transgression, predominantly through flood alluviation, and often manifest as river valley plains, river terraces, and other landforms. These strata are composed of well-sorted and rounded sand and gravel, with a relatively loose structure and a thickness ranging from 2 to 12 meters. The geographical location of the selected area is shown in Fig 2.

### Evaluation factors

**Fig 3:**
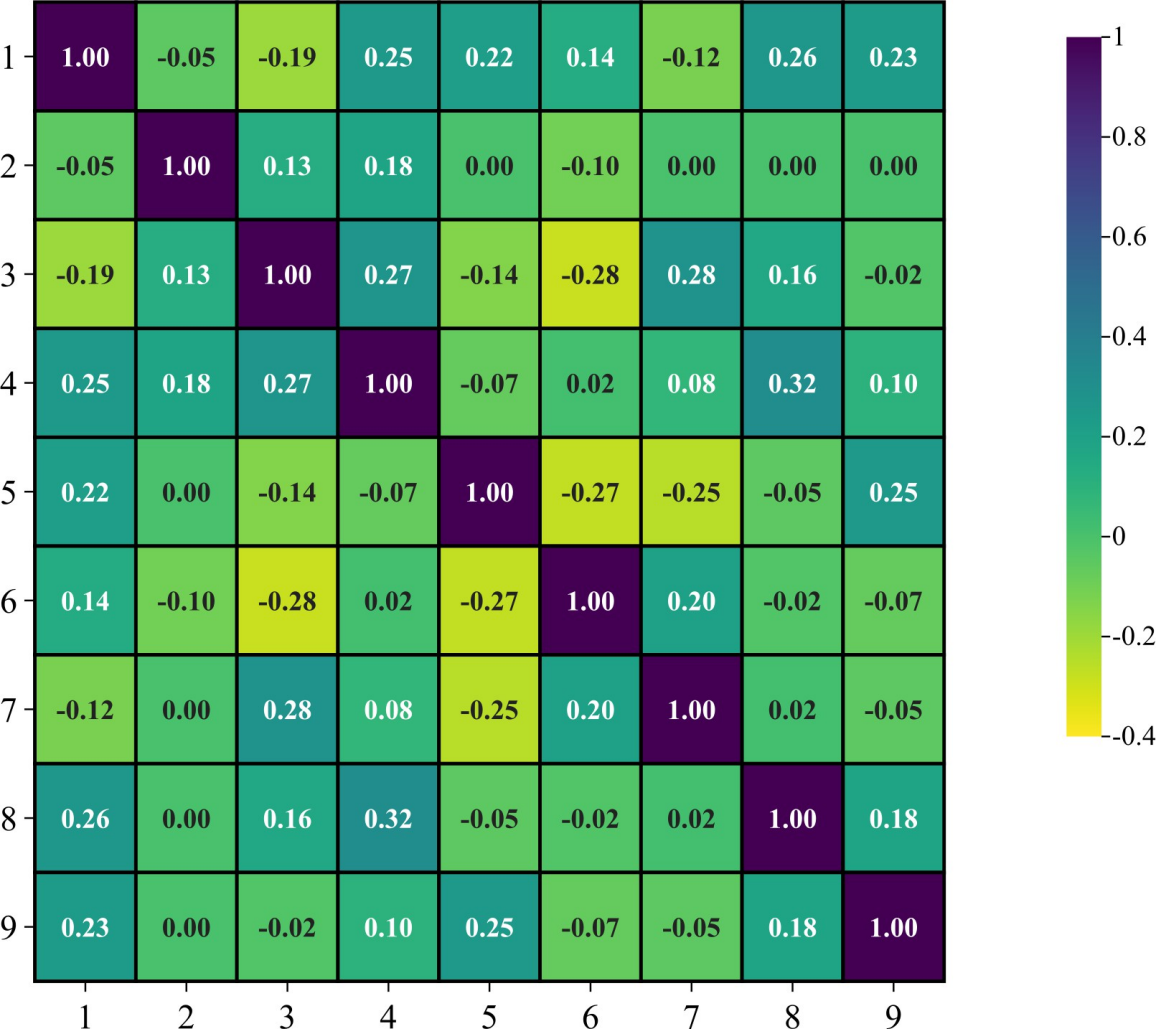
Correlation heatmap of evaluation factors; (1): The thickness of the surface fill layer; (2): Distance to underground rivers and blind ditches; (3): Burial depth of the top layer of saturated silty sand soil; (4): The density of the drainage pipe network; (5): Burial depth of the underground confined water level; (6): Rainfall (weekly) (2022); (7): The thickness of soft soil layer; (8): Groundwater richness; (9): Ground subsidence rate (2022).

**Fig 4:**
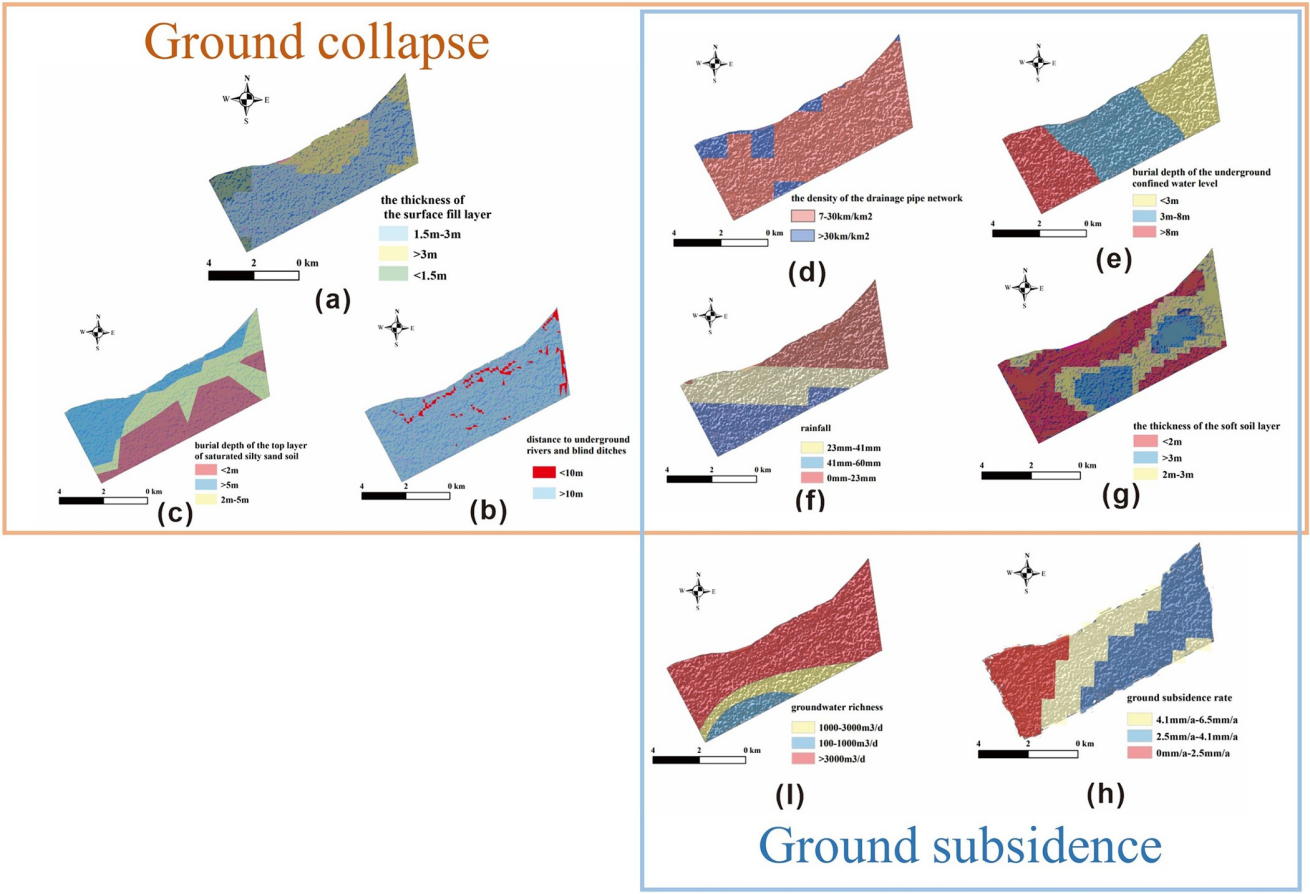
Evaluation factors of ground deformation disasters.

Given the intricate nature of urban geological environments and the distinct aspects of ground deformation disasters, it’s critical to choose evaluation factors that thoroughly account for the intensity of human activities within the study area. Additionally, these factors should be analyzed deeply in combination with the geological and cultural features of the region. This comprehensive approach ensures that assessments are not only accurate but also reflective of the diverse influences impacting the area’s susceptibility to geological hazards. To determine which influencing factors are most associated with urban ground deformation disasters in Hangzhou, we collaborated with experts from the Hangzhou urban geological safety assessment at the Zhejiang Geological Survey. Based on field inspections and expert analyses, ground collapses in the study area are primarily due to poor soil properties and damage to pipelines caused by groundwater factors. Meanwhile, ground subsidence is mainly due to the study area being located in a plain, with the distribution of soft soil layers, human activities, and hydrological factors being the primary reasons for ground subsidence occurrences. Given these factors, we identified 9 key factors as preliminary evaluation indicators for urban ground deformation disasters and conducted a correlation analysis to prevent issues such as overfitting or frequent misjudgments in the machine learning process, as depicted in the correlation heatmap (Fig 3). The evaluation factors used for the disaster, data sources, and data attributes are detailed in Table 1. For detailed maps of each evaluation factor, please refer to Fig 4.

**Table 1:** Evaluation factors information.

Factors	Disasters	Sources	file type
Groundwater richness	Ground subsidence	Zhejiang Geological Survey	Shapefile
Burial depth of the top layer of saturated silty sand soil	Ground collapse	China Geology Survey, Nanjing Center	Raster Cell Size (X, Y) (32,32)
Burial depth of the underground confined water level	Both	China Geology Survey, Nanjing Center	Shapefile
The thickness of the surface fill layer	Ground collapse	China Geology Survey, Nanjing Center	Raster Cell Size (X, Y) (32,32)
Rainfall (weekly) (2022)	Both	Zhejiang Geological Survey	Raster Cell Size (X, Y) (32,32)
The thickness of soft soil layer	Both	China Geology Survey, Nanjing Center	Raster Cell Size (X, Y) (32,32)
Ground subsidence rate (2022)	Ground subsidence	Zhejiang Geological Survey	Raster Cell Size (X, Y) (32,32)
The density of the drainage pipe network	Both	Zhejiang Geological Survey	Raster Cell Size (X, Y) (32,32)
Distance to underground rivers and blind ditches	Ground collapse	Zhejiang Geological Survey	Shapefile

### Declaration of generative AI and AI-assisted technologies in the writing process

During the preparation of this work, the author(s) used ChatGPT-4 to assist in comprehensive ground disaster assessment by substituting human expert judgement with the AI’s capabilities in weight determination. This process involved ChatGPT-4’s analysis, judgement, and rationale provision based on textual data. After using this tool/service, the author(s) reviewed and edited the content as needed and take(s) full responsibility for the content of the publication.

### Participant recruitment

The recruitment of experts was conducted by the Nanjing Center of the China Geological Survey during the completion of the Hangzhou Multi-Factor Geological Survey Project (DD20190281), in collaboration with the Zhejiang Provincial Geological Bureau. The experts involved are well-acquainted with the situation in Hangzhou. As part of their tasks, the experts were required to use the table in Fig 11 of the paper to assign scores, which involved the use of the Analytic Hierarchy Process (AHP). The cooperation with the experts was based on the project requirements, specifically the needs of the work. Besides participating in scoring the table, the experts did not take part in any other aspects of the paper.

### Random forest—back propagation neural network coupling model

To improve the precision of models predicting ground collapse and mitigate the challenges posed by the limited size of disaster zones, which often results in a scarcity of data points, we recommend employing a hybrid model combining Random Forest with Back Propagation Neural Network. Random Forest utilizes a collective method within machine learning, consisting of multiple decision trees. Each tree operates on a randomized segment of the dataset and its features, which boosts the overall accuracy and robustness of the model. This technique is particularly adept at processing varied inputs and identifying subtle patterns. To clarify how Random Forest operates, we can depict its ensemble technique through specific equations, enhancing comprehension of its methodological framework:

$$Variance\;Reduction=Var(S)-(\frac{\left|S_{left}\right|}{\left|S\right|}Var(S_{left})+\frac{\left|S_{right}\right|}{\left|S\right|}Var(S_{right}))$$ (1)

Where *Var*(*S*) represents the variance of the target variable in the entire dataset *S*, |*S*_*left*_| and |*S*_*right*_| denote the number of samples in the left and right subsets post-split, respectively. Eq (1) highlights how Random Forest effectively reduces overfitting by incorporating diverse data samples.


$$G(S)=1-\sum_{i=1}^{n}p_{i}^{2}$$ (2)

Where $p_{i}^{2}$ indicates the proportion of the samples in set *S* that belong to class *i*, and *n* is the total number of classes. This measure is critical in determining the best split at each node within the trees. The Eq (2) not only allows Random Forest to handle a variety of input types effectively but also enhances its capability to detect subtle patterns in complex datasets.

On the other hand, the Backpropagation Neural Network stands out as a fundamental element in machine learning, especially effective for tasks where linear methods fall short. It consists of several layers, each contributing to the network’s ability to refine its predictions through continuous adjustments to its parameters (weights and biases). To provide a clearer insight into the learning mechanism of a Backpropagation Neural Network, we can examine specific equations that detail this iterative process:

$$W_{ij}^{\left(new\right)}=W_{ij}^{\left(old\right)}-\eta \frac{\partial L}{\partial W_{ij}}$$ (3)

Where $W_{ij}^{\left(old\right)}$ and $W_{ij}^{\left(new\right)}$ are the old and new values of the weight between nodes *i* and *j*, *η* is the learning rate, and $\frac{\partial L}{\partial W_{ij}}$ represents the gradient of the loss function ℒ with respect to the weight $W_{ij}^{\left(new\right)}$ Eq (3) delineates the core mechanism by which the network learns by iteratively adjusting its weights.


$$\sigma (x)=\frac{1}{1+e^{-x}}$$ (4)

The *σ*(*x*) function, known as the sigmoid activation function, is essential in neural networks for normalizing inputs x to an output range between 0 and 1. This transformation is crucial as it allows the network to manage non-linear relationships in the data effectively. Eq (4) demonstrates how the sigmoid function converts linear inputs into bounded outputs, enhancing the network’s capability to address probabilistic decisions and complex non-linear phenomena.

**Fig 5:**
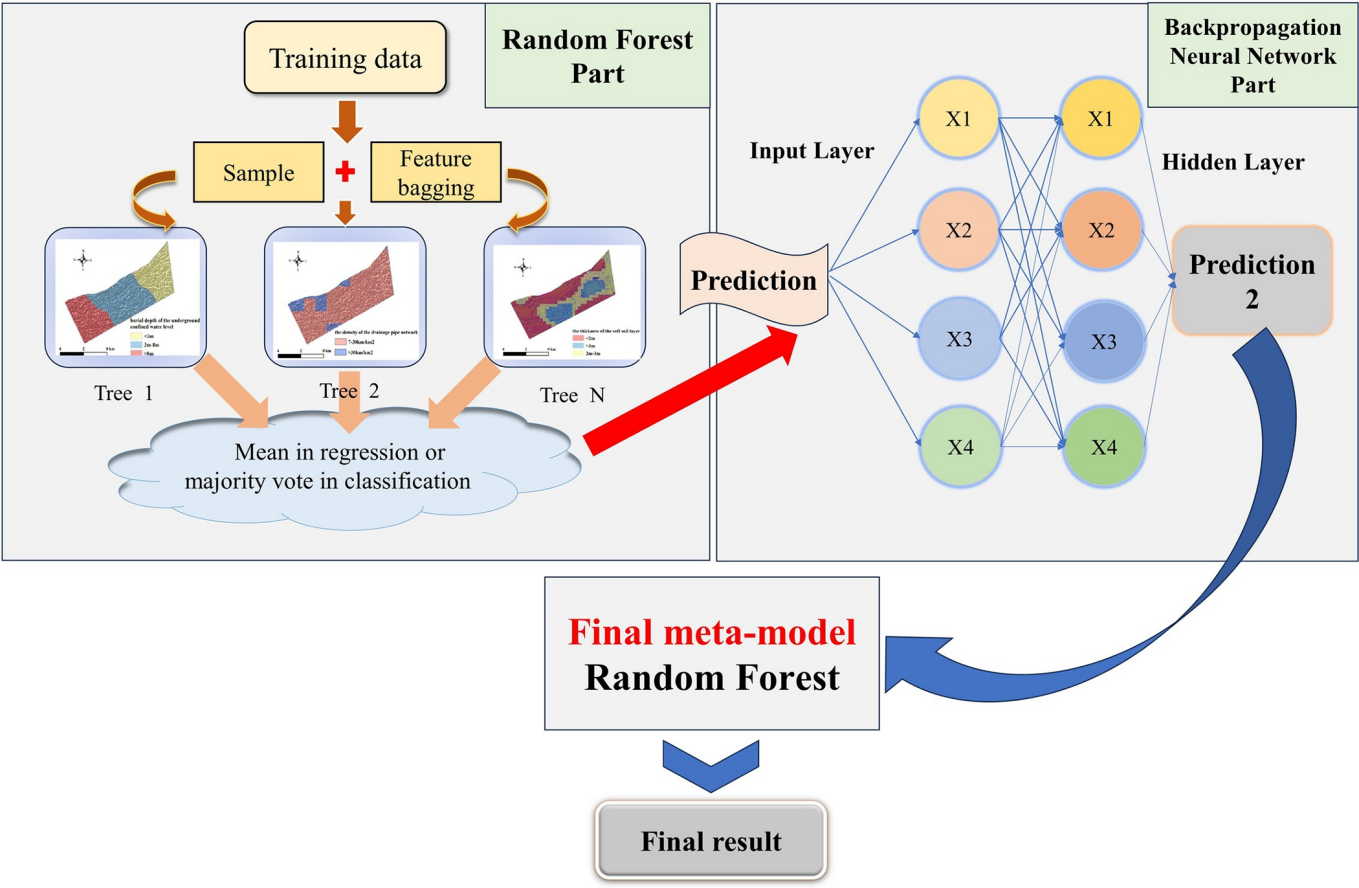
Schematic diagram of the structure for the RF-BP neural network coupling model.

Integrating the Random Forest and Backpropagation Neural Network results in a powerful, synergistic model. The Random Forest begins the process by managing diverse inputs and laying the groundwork for the analysis. In our implementation, we have established two foundational models and a stacking classifier: The Random Forest Classifier includes 100 trees and uses a random seed of 42 to ensure consistency; the Neural Network Classifier features two hidden layers with 100 and 50 neurons, respectively, operates up to 1000 iterations, and maintains the seed of 42 for repeatability. The Stacking Classifier then leverages these models as its base, combining their predictions with an additional Random Forest of 100 trees serving as the final meta-model to solidify the decision-making process. This layered approach allows the Backpropagation Neural Network to further refine the analysis, enhancing the overall model’s capability to assimilate extensive data, distill key insights, and deliver accurate predictions. The operational framework of this ensemble model is illustrated in Fig 5.

## Results

### Susceptibility assessment

In this research, we utilized the "Raster to Point" function in ArcGIS to convert 27,898 data points for ground subsidence susceptibility assessment, where susceptible regions were identified based on regional cumulative subsidence data. For accurate model training, 70% of the data were randomly selected for the training set via Python, ensuring robustness in ground subsidence susceptibility evaluation. For ground collapse susceptibility, we maintained a balanced approach by using a 1:1 ratio of disaster to non-disaster points in the training set (with disaster points marked as 1 and non-disaster as 0). Given the limited extent of collapse areas, we obtained 300 collapse data points and chose 210 points each from collapse and non-collapse categories for training. This strategy mitigates bias towards more frequently occurring non-collapse conditions.

The trained models were then applied to predict the likelihood of ground collapse for each pixel (with probabilities between 0–1) and to estimate the cumulative subsidence for each location. Post-simulation, model effectiveness was gauged using the Area Under the ROC Curve (AUC). The standalone Random Forest model achieved an AUC of 0.83 for ground collapse susceptibility. However, the combined Random Forest and Back Propagation Neural Network (RF-BPNN) model enhanced the AUC to 0.89, reflecting a 7% accuracy improvement. For cumulative subsidence prediction, the RF-BPNN model exhibited high accuracy with AUC scores of 0.99 for low (less than 24mm), 0.99 for moderate (24mm-64mm), 0.97 for high (64mm-96mm), and 0.99 for very high (96mm-128mm) susceptibility levels.

**Fig 6:**
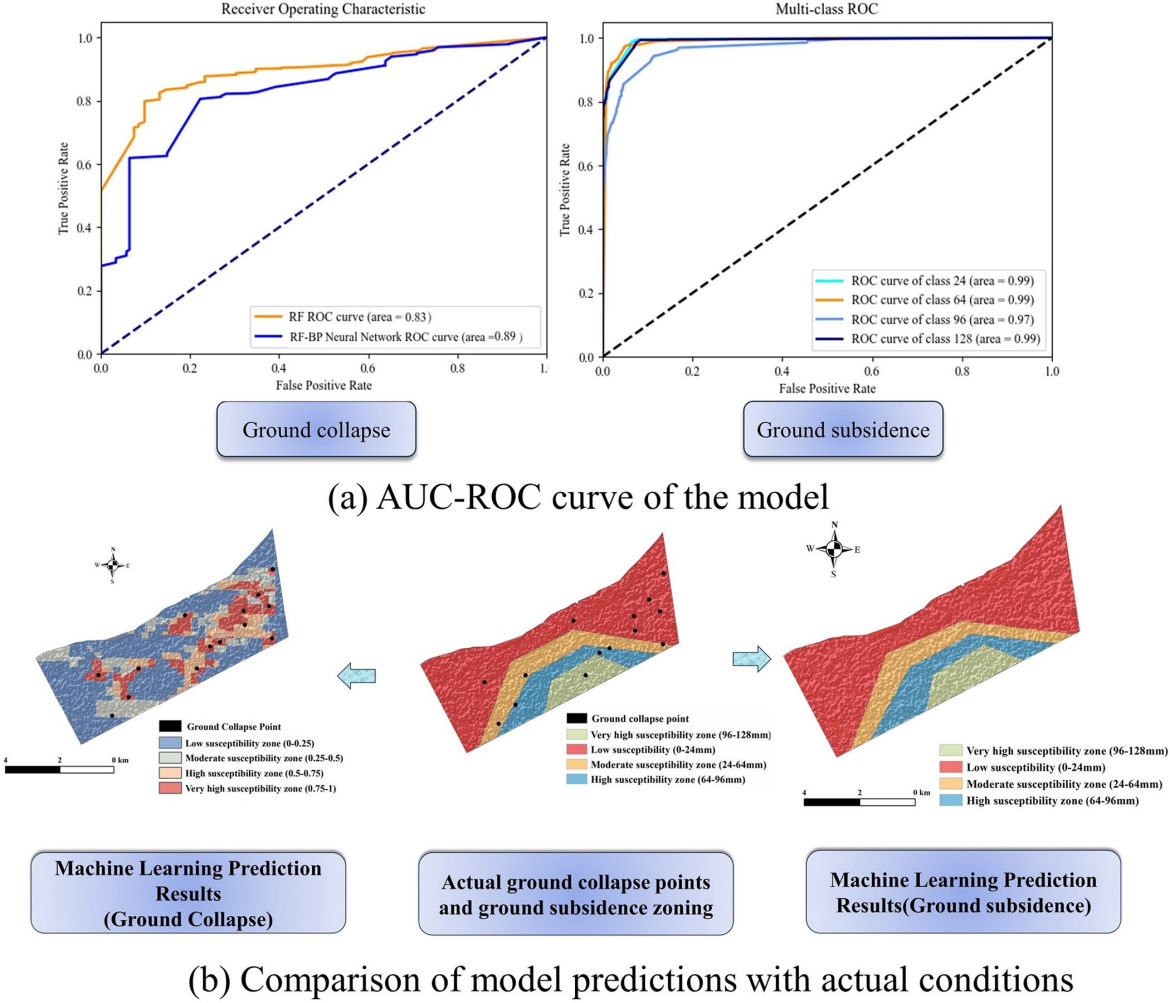
(a)AUC-ROC curve (b) Comparison of model predictions with actual conditions.

Post-prediction, the results of the RF-BPNN model were integrated into ArcGIS 10.8 to delineate ground deformation disaster susceptibility zones. Fig 6B illustrates a comparison of the actual ground deformation scenarios from 2018–2022, sourced from Zhejiang Geological Survey shapefile data and analyzed using InSAR techniques, with susceptibility zones expertly demarcated. Ground collapse probabilities were categorized into four levels: low (0–0.25), medium (0.25–0.5), high (0.5–0.75), and very high (0.75–1) susceptibility. Table 2 details the zoning area percentages and disaster point distributions.

**Table 2:** Susceptibility zone for ground collapse disasters in the study area.

Susceptibility level	Area /km^2^	Percentage /%	Number of disaster points	Percentage of total disaster points/%
**Low susceptibility**	14.16	44.31%	0	0
**Moderate**	7.33	22.94%	1	7.41
**High**	4.59	14.35%	1	7.41
**Very high**	5.89	18.40%	12	85.71

For ground subsidence, predicted maps were juxtaposed with actual monitored data, categorizing susceptibility into four intervals: low, medium, high, and very high. The overlap between simulated and actual cumulative subsidence data was quantitatively impressive at 90.31%, with specific overlap percentages listed in Table 3 for each category. These findings underscore the robustness of the model in closely mirroring actual conditions in the study area, affirming its strong performance and reliability in disaster susceptibility assessments.

**Table 3:** Comparison of overlap degrees between monitored data and predicted data.

Ground subsidence	Monitoring results (points)	Model prediction (points)	Overlap degree
**0mm-24mm**	9069	10157	89.29%
**24mm-64mm**	11064	11437	96.74%
**64mm-96mm**	5090	4376	85.97%
**96mm-128mm**	6902	6161	89.26%

### Assessment based on ChatGPT4

**Fig 7:**
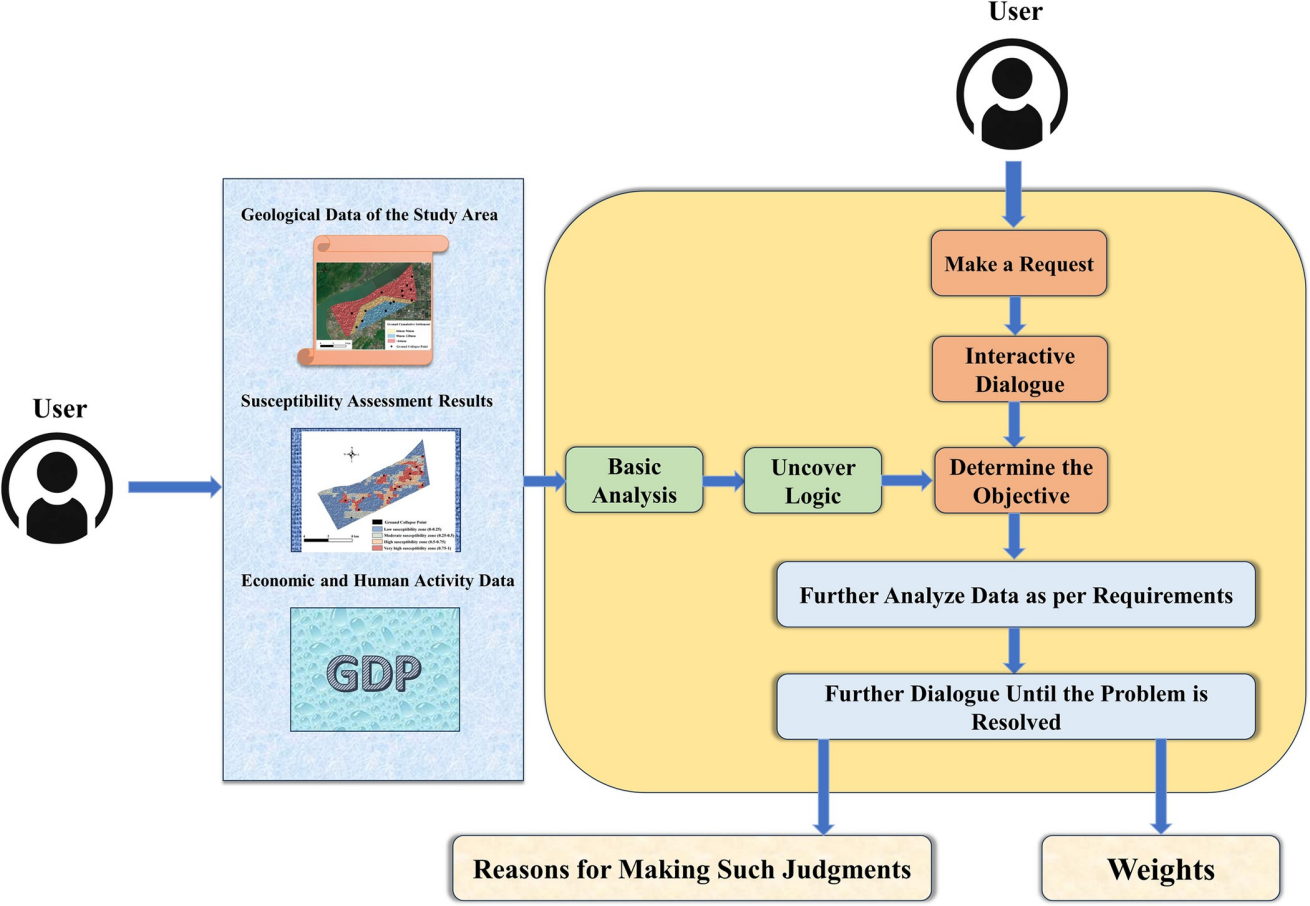
The principle of judgment by large language models.

Large language models (LLMs) are AI models crafted to comprehend and produce human language. Trained with extensive text data, they excel in various tasks such as text summarization, translation, sentiment analysis, among others. A distinctive feature of LLMs is their immense scale, encompassing billions of parameters to learn intricate language patterns. These models are generally based on deep learning architectures, such as transformers, facilitating impressive performance in diverse NLP tasks. In this study, the LLM employed is ChatGPT-4, developed by OpenAI. ChatGPT-4, an advanced AI language model, is widely utilized across various industries for its superior language comprehension and generation abilities, comprehensive knowledge base, creativity, programming support, and image processing capabilities. In this research, ChatGPT-4 was supplied with documents akin to those provided to human experts, including basic geological information of the study area, historical ground deformation incidents, their economic and human safety impacts, and prior susceptibility evaluation content. Utilizing ChatGPT-4’s data parsing capabilities, it performs fundamental analyses. Throughout this process, ChatGPT-4 employs logical reasoning to comprehend how various factors, including geological features and historical disasters, affect the weighting of ground collapse and subsidence, as well as the correlations and potential impacts among the data. Subsequently, it leverages its extensive pre-trained knowledge base for data analysis. ChatGPT-4 can provide insights by applying its understanding of analogous situations, particularly in interpreting geological events and disaster impacts. Researchers can initiate interactive dialogues to pose additional questions or request more in-depth analysis of specific data. This interactive capability renders the analysis process more flexible and adaptable. Ultimately, ChatGPT-4 renders judgments and offers rational explanations. The fundamental logic of this process is depicted in Fig 7.

This study utilizes ChatGPT-4, and it is declared that its use adheres to ChatGPT-4’s terms of service, respects copyright, ensures content appropriateness, avoids personal privacy infringement, and refrains from generating false information. Additionally, human experts fully monitored the AI’s predictive process in this case, and the data analysis results underwent human review and analysis.

**Fig 8:**
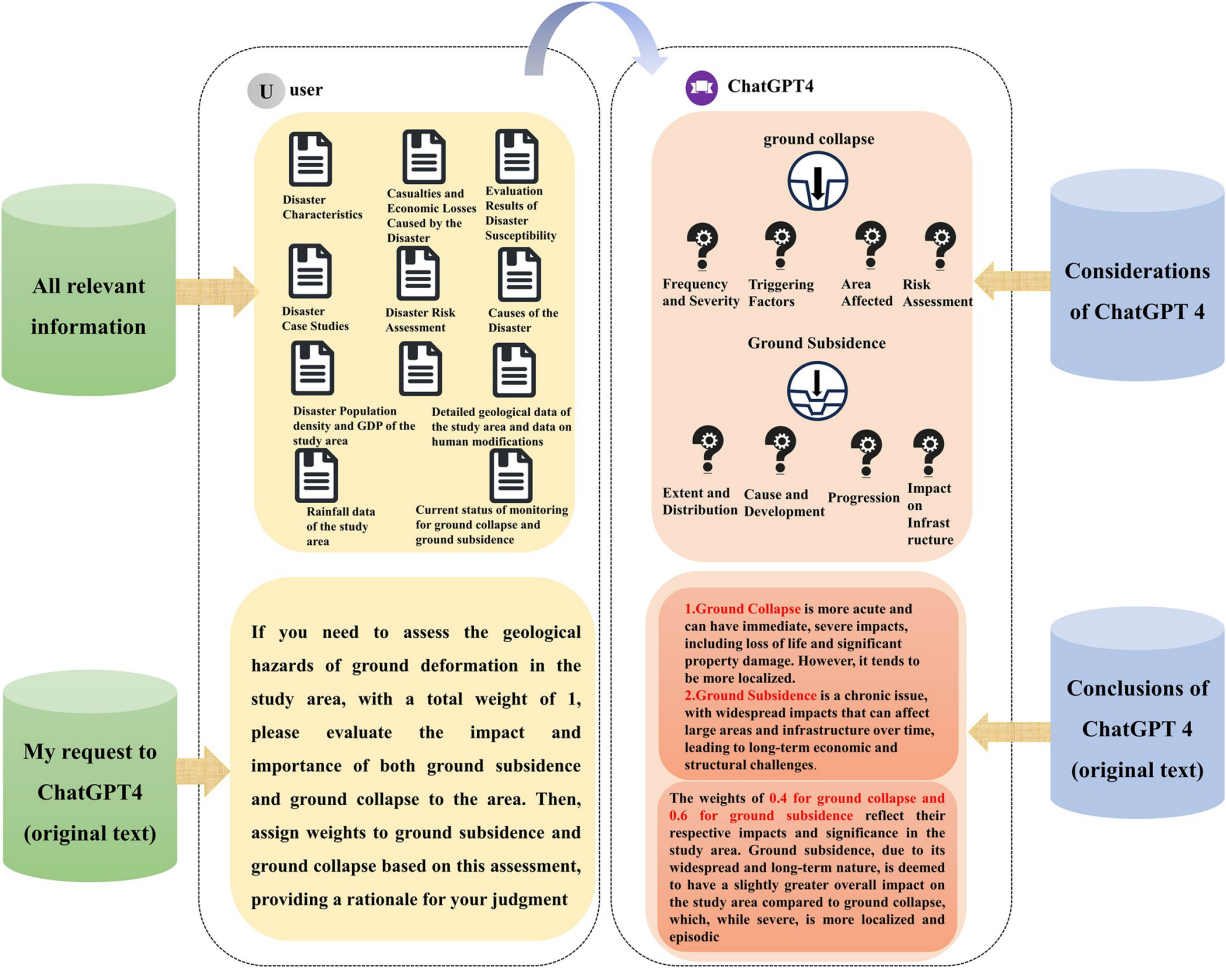
The process of judgment by large language models in this study.

In this research, the interaction with ChatGPT-4 commenced with organizing and directly uploading the necessary textual materials from Hangzhou geological experts to ChatGPT-4. As the research materials were exclusively in text format, ChatGPT- 4’s image reading comprehension capabilities were not utilized in this process. Subsequently, ChatGPT-4 was requested to analyze the data as a geological expert and make judgments on the weights of ground collapse and subsidence, elucidating the basis for these judgments. Once ChatGPT-4 fully comprehended the document materials’ logic and the researchers’ requests, it conducted the analysis and provided weight judgments, as detailed in Fig 8.

## Discussion

**Fig 9:**
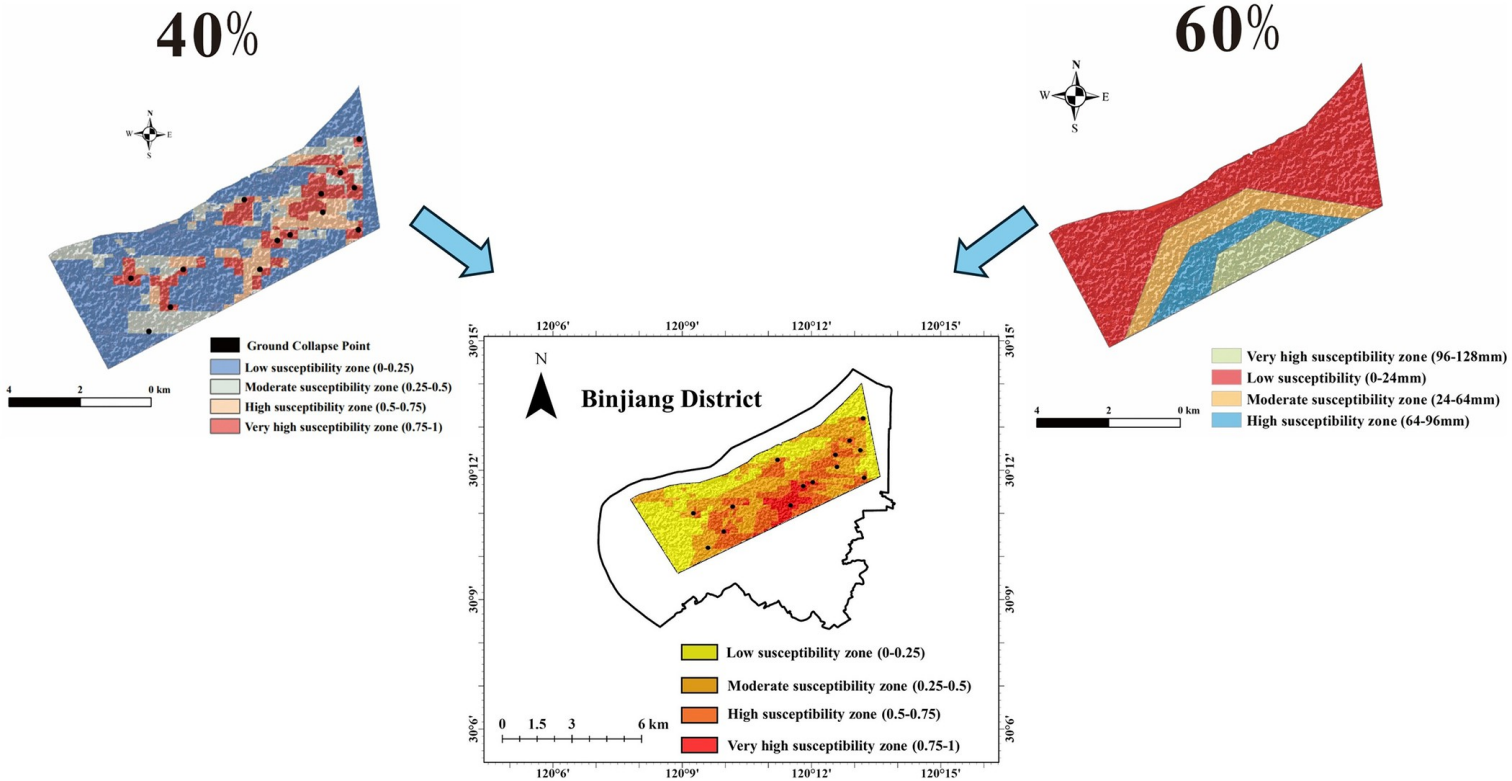
Comprehensive assessment of ground deformation hazard susceptibility in the study area.

In the comprehensive assessment of ground deformation hazards, with a weighting of 0.4 for ground subsidence and 0.6 for ground collapse, we categorized the risk zones as follows: low susceptibility zone (0–0.25), moderate susceptibility zone (0.25–0.5), high susceptibility zone (0.5–0.75), and very high susceptibility zone (0.75–1). To create the comprehensive susceptibility assessment map, we utilized the raster calculator function in ArcGIS 10.8 software, resulting in the overlay of single-hazard susceptibility maps for ground collapse and ground subsidence, as depicted in Fig 9. The proportions in this map are as follows: low susceptibility zone 23.71%, moderate susceptibility zone 42.61%, high susceptibility zone 27.16%, and very high susceptibility zone 6.52%.

Based on the research area data, we have observed that the high and very high susceptibility zones in the evaluation results feature elevated bridges and are densely populated areas with significant human activity. Additionally, these areas exhibit a higher prevalence of high-rise buildings, increased utilization of underground space, reduced thickness of surface fill, and a dense distribution of underground pipelines. In contrast, the moderate and low susceptibility zones in the evaluation results are situated in proximity to the Qiantang River, characterized by fewer buildings and pipelines, as well as relatively minor human activity alteration.

However, due to the imbalance in the proportion of collapse disaster sites versus non-disaster sites, the evaluation results of machine learning models tend to overly rely on the disaster sites rather than the evaluation factors, even under the premise of a high AUC value. This highlights a limitation in using machine learning models for susceptibility assessment in small-scale geological disasters. Therefore, to enhance application and research in this area, we will increasingly integrate field investigations to achieve more accurate predictive outcomes.

To validate the accuracy of ChatGPT-4’s judgments, specifically its ability to match the current situation of the study area using provided disaster materials and questions, experts from the Zhejiang Geological Survey were invited to employ the same materials and utilize the Analytic Hierarchy Process (AHP). This process entails decomposing decision-making elements into hierarchies, including objectives, criteria, and alternatives, followed by qualitative and quantitative analysis to determine the weights of ground deformation disasters in the study area.

**Fig 10:**
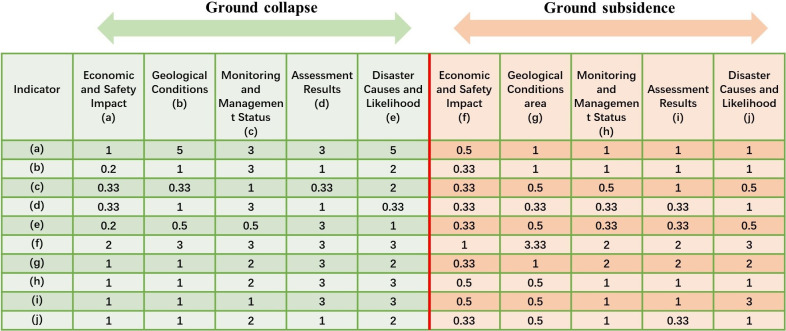
APH assessment result.

During the AHP judgment process, experts received the same materials as ChatGPT-4 to base their judgments upon. The objective layer in the judgment process focused on the weight values of ground collapse and subsidence, while the criteria layer comprised ten indicators, including the impacts on the economy and human safety, relationship with geological conditions, current monitoring and management status, susceptibility assessment results, and causes and probabilities of occurrence. Of these, the first five indicators were weight determinants for ground collapse, and the latter five pertained to ground subsidence. Finally, by summing the weights of the disaster indicators for ground collapse and subsidence, the overall weight of the disaster was ascertained, leading to an aggregate judgment on the weights of ground deformation disaster indicators. The specific AHP judgment table and disaster weight table are depicted in Fig 10 and Table 4.

**Table 4:** Susceptibility zone for ground collapse disasters in the study area.

Indicator		Eigenvector	Weights (%)	Total weight (%)
Ground collapse	Economic and Safety Impact	1.477	14.772	38.768
	Geological Conditions	0.833	8.331	
	Monitoring and Management Status	0.519	5.195	
	Assessment Results	0.553	5.532	
	Disaster Causes and Likelihood	0.494	4.938	
Ground subsidence	Economic and Safety Impact	2.075	20.753	61.232
	Geological Conditions	1.222	12.217	
	Monitoring and Management Status	0.988	9.883	
	Assessment Results	1.1	11.001	
	Disaster Causes and Likelihood	0.738	7.378	

**Fig 11:**
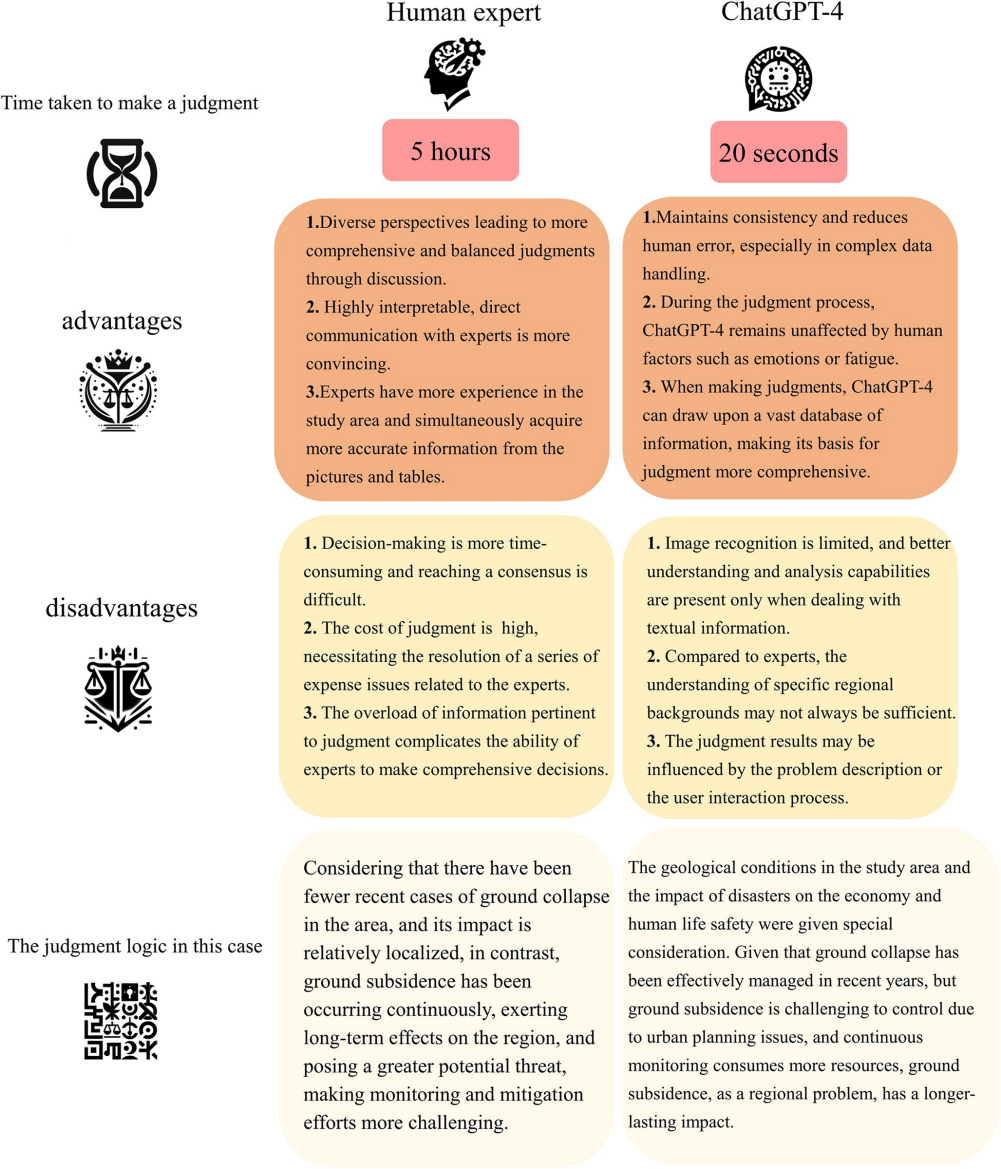
The decision-making logic and the advantages and disadvantages of human experts compared to ChatGPT-4.

According to the judgment results obtained by experts using the Analytic Hierarchy Process (AHP) based on the same data as ChatGPT-4, the weight ratio of ground collapse to ground subsidence is 0.39:0.61. This is remarkably similar to ChatGPT-4’s judgment result of 0.4:0.6. Additionally, upon comparing the judgment processes of ChatGPT-4 and experts, it can be inferred that ChatGPT-4 possesses distinct advantages over expert manual judgment regarding judgment logic and reason explanation. However, despite the similarities in the considerations of ChatGPT-4 and experts, there are still some immature aspects present in ChatGPT-4’s performance. The specific comparison can be observed in Fig 11.

## Conclusion

1)This study explores regions characterized by filled and silty sand in Hangzhou, addressing the gap in utilizing machine learning for the assessment of urban ground deformation susceptibility. Through correlation analysis, we identified nine key factors to construct a Python-based RF-BP neural network ensemble model. This model effectively combines the robustness of random forests in handling multiple variables and detecting nonlinear patterns with the precision of the BP neural network in improving predictions.

2)Following the generation of ground collapse and subsidence susceptibility maps, we employed the advanced Language Model LLM ChatGPT-4 to automate the disaster weighting process, a task conventionally undertaken by experts. Given area-specific disaster data, ChatGPT-4 effectively assumed the role of a geologist and derived a weight ratio of 0.4:0.6 for ground collapse versus subsidence. Expert validation through the Analytic Hierarchy Process (AHP) substantiated ChatGPT-4’s rationale, underscoring its effectiveness in geological analysis.

3)The study highlights the advantages of ChatGPT-4 in analysis, particularly its speed, objectivity, and reliance on a database-driven approach. Nevertheless, we have identified certain limitations, including restricted image recognition, insufficient explainability, and a lack of field-specific expertise. Despite extensive data input, certain aspects remain unattended to, indicating that the full analytical and decision-making potential of ChatGPT-4 warrants further exploration. The ongoing development of Large Language Models (LLMs) like ChatGPT-4 presents an auspicious avenue for future geological research.

It should be noted that there are significant challenges in maturely applying large language models (LLMs) like ChatGPT-4 to decision-making, particularly due to the "black box" nature of their credibility and decision outcomes. The rationale provided by ChatGPT-4 often remains obscured in a black box process. Therefore, when applying judgments based on language models to practical scenarios, it is crucial to ensure that the conclusions align with the actual geological conditions. Additionally, final judgments should ideally be validated by geological experts, positioning LLM support as an auxiliary tool rather than a replacement for human expertise. We also reiterate our call for more geologists to experiment with LLMs in real case studies, to further explore the possibilities of this approach and aid its continuous improvement.
